# Use of Artificial Intelligence for Medical Literature Search: Randomized Controlled Trial Using the Hackathon Format

**DOI:** 10.2196/16606

**Published:** 2020-03-30

**Authors:** Dominik Schoeb, Rodrigo Suarez-Ibarrola, Simon Hein, Franz Friedrich Dressler, Fabian Adams, Daniel Schlager, Arkadiusz Miernik

**Affiliations:** 1 Medical Center – Department of Urology Faculty of Medicine University of Freiburg Freiburg Germany

**Keywords:** artificial intelligence, literature review, medical information technology

## Abstract

**Background:**

Mapping out the research landscape around a project is often time consuming and difficult.

**Objective:**

This study evaluates a commercial artificial intelligence (AI) search engine (IRIS.AI) for its applicability in an automated literature search on a specific medical topic.

**Methods:**

To evaluate the AI search engine in a standardized manner, the concept of a science hackathon was applied. Three groups of researchers were tasked with performing a literature search on a clearly defined scientific project. All participants had a high level of expertise for this specific field of research. Two groups were given access to the AI search engine IRIS.AI. All groups were given the same amount of time for their search and were instructed to document their results. Search results were summarized and ranked according to a predetermined scoring system.

**Results:**

The final scoring awarded 49 and 39 points out of 60 to AI groups 1 and 2, respectively, and the control group received 46 points. A total of 20 scientific studies with high relevance were identified, and 5 highly relevant studies (“spot on”) were reported by each group.

**Conclusions:**

AI technology is a promising approach to facilitate literature searches and the management of medical libraries. In this study, however, the application of AI technology lead to a more focused literature search without a significant improvement in the number of results.

## Introduction

Mapping out the research landscape around a project is time consuming and often frustrating, as the sheer size of published data is often impossible to read and understand, much less be put in context. To overcome these challenges, the current scientific standard is a systematic literature review using standardized methodology in accordance with the PRISMA (Preferred Reporting Items for Systematic Reviews and Meta-Analysis) guidelines [1] and the Cochrane regulations [2]. This process is thorough and reliable; however, it also requires a significant amount of time and resources. To overcome these limitations and make literature searches more effective, a promising approach is the application of artificial intelligence (AI) technology, which can screen vast amounts of data faster and more efficiently than a human researcher. When founded in 2016, the IRIS.AI search engine was the first application addressing the problem of screening vast amounts of scientific literature using AI. Other promising formats have emerged since then, like Google's new AI service Talk to Books [3]; however, IRIS.AI is still the only application that exclusively caters to the scientific field.

The current topics of interest for our research group are virtual reality (VR) and augmented reality (AR) in surgery. VR and AR are new technologies with a high potential for application in the medical field. Besides use in diagnostic imaging applications and the facilitation of minimally invasive procedures, the implementation of these technologies can substantially improve medical education, particularly when it comes to teaching practical tasks. Many studies and projects have been conducted on this topic; however, more research and development work is necessary to routinely implement those technologies into medical education. To investigate the most promising approaches and questions pertaining to the implementation of VR and AR, intensive literature research is necessary. The intention of this study was to evaluate the commercially available AI search engine IRIS.AI in the application of medical literature research on these topics.

## Methods

### Study Design

To evaluate the AI search engine, we gathered surgery experts from different specialties (ie, general surgery; ear, nose and throat surgery; neurosurgery; orthopedics and trauma surgery; and urology), information technology (IT) experts, and medical engineers to perform a systematic literature search on a specific scientific question in a predefined time frame. Participants were randomized to three different teams. All literature research was done in a 1-day event, and the three teams competed for the best overall result. All participants were provided similar computers for the study, and all search activities were performed at the same location. Communication between the different teams was not permitted. This format, called a “science hackathon” by IRIS.AI, was originally applied in the IT industry with the goal of finding a solution to a programming challenge. This format has been adapted for multiple fields, including scientific purposes and general topics such as marketing [4].

### Group Participants

A total of 17 scientists with expertise in medical education, surgery, IT, or engineering took part in the literature research. One participant cancelled on the day of the event and prevented an even distribution of group members. All scientists had an extensive background in scientific research and, therefore, a high proficiency in literature research using scientific databases. We recruited participants through public announcements and information given to collaborating research groups and institutes (Multimedia Appendix 1). All participants of this study consented to participate. The need for ethical approval was waived since no personal data was used in this study. The participants were divided according to their field of expertise to ensure an even distribution of medical and engineering experts. They were then randomly assigned to one of three groups. A detailed description of the group members’ scientific backgrounds can be found in Multimedia Appendix 2. This resulted in two groups with 6 members and one group with 5 members. After the groups were determined, one was randomly designated as the control group without access to AI technology.

### Literature Search

**Figure 1:**
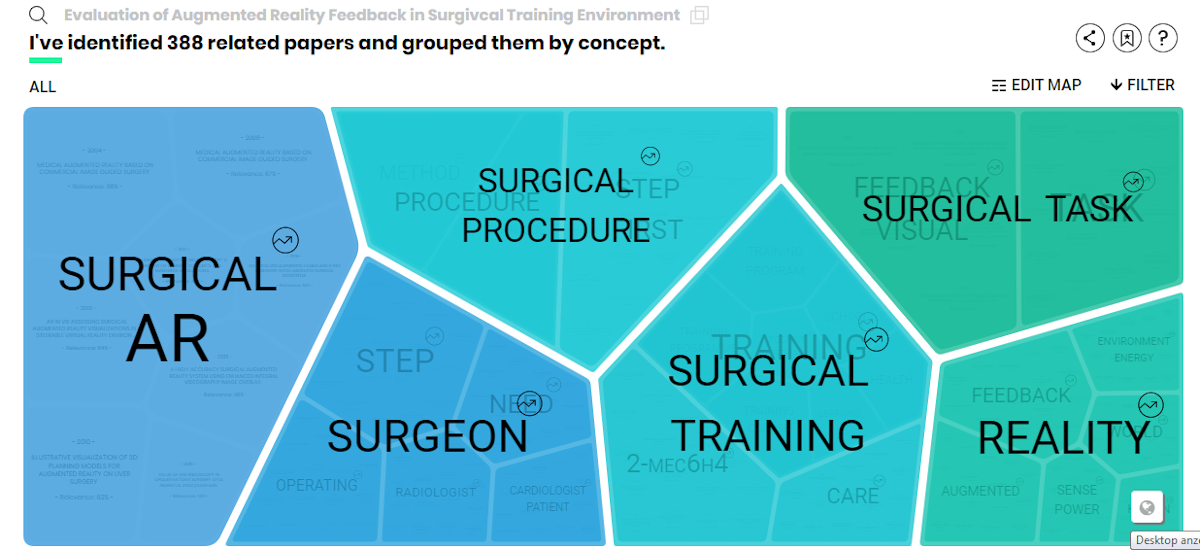
Screenshot of a search result “map” created by the IRIS.AI artificial intelligence research assistant. AR: augmented reality.

The IRIS.AI search engine can understand key concepts of a given scientific article and can search for relevant data that is similar or connected to the article by using an AI algorithm. The search results are displayed in a visual map divided in subcategories related to the main topic (Figure 1). A detailed description on how the AI software operates can be found at the website of the providing company (https://the.iris.ai/). Research time was limited to 5 hours and divided into two research sessions. All groups were instructed to document their research results in a standardized format using a report form (Multimedia Appendix 3), which was issued prior to the commencement of the research time. In addition, searches were monitored using a key-logging program on the search computers, and remote surveillance was used on the PC screen. The control group used Google Scholar, Web of Science, and PubMed in their literature research.

### Definition of Research Task

All participants were issued a written problem statement at the beginning of the event with a clearly defined research task (Multimedia Appendix 4). The general question was: “What research and development work is necessary to build a ‘ready to use’ adaptive augmented reality (AR) system for surgeons to teach surgical residents how to perform a surgical procedure?” The three groups were asked to focus their literature search on the following three main problems defined by the posed question: (1) the recognition of reality such as anatomy or surgical instruments, which is necessary to fulfill the task of supporting a surgical resident; (2) the hardware necessary to provide the proposed system such as voice guidance or AR glasses; and (3) the pedagogical concept behind an AR system that “teaches” a surgeon how to perform a surgical procedure “step-by-step”. The researchers were asked to find literature on the potential solutions to these problems, the application of similar approaches, and the capability of available technologies. Additionally, they were asked to find conceptual studies on how to combine available technologies.

### Documentation of Results

Before the research time ended, all groups were asked to document their research results using a standardized report form (Multimedia Appendix 3). In addition, the groups were supposed to differentiate between the three main problems and sort their results on the report form accordingly.

### Final Evaluation

A committee of three experts with backgrounds related to the posed scientific question evaluated and ranked the search results according to scientific quality and quantity criteria using a standardized scoring sheet (Multimedia Appendix 5). The experts included a urological surgeon with experience in medical and surgical education, a software engineer with specific background in the field of AR technology, and an engineer in charge of research and development at a large medical technology company. The results of the quantitative and qualitative evaluation were added to a final score, and teams were ranked accordingly (Multimedia Appendix 6).

## Results

All participating teams were able to detect literature relevant to the topic. The first team, using the IRIS.AI search tool, listed 13 relevant papers on their score sheet, which were all judged as relevant for the proposed topic. The quality of the found studies was ranked very high, with a total score of 27. Multiple studies applying AR in surgical fields were detected, which shows that AR can provide an accurate visual representation of anatomical structures and can be a helpful tool during surgical procedures [5-10]. The second team supported by AI listed 15 relevant papers, but only 8 were found as related to the field by the judges. The quality of the ranked articles was awarded with 19 points. The literature review performed by this team followed a problem-oriented strategy that focused directly on researching the challenges proposed by the scientific question and the potential solutions available in the literature. Regarding the perception of reality and the tracking of deformable objects, available technology was detected, which technically addresses the challenges of an adaptive AR system for intraoperative guidance, but the necessity of high computational power and the limited reliability are still challenges that need to be addressed before a clinical use is possible [11-13]. The control group listed the highest number of results with 46 identified scientific studies, but only 10 studies were found to be related to the field. The quality of the studies was graded with a total of 25 points. This group provided a broad selection of literature with an overview of the capabilities of existing technologies for intraoperative support of the operating surgeon, as well as the limitations and problems that need to be addressed when implementing the proposed system into clinical practice. In total, 20 relevant scientific studies were identified throughout the event covering all aspects of the question. Of those 20 studies, the AI groups contributed 7 findings each, while the control group contributed 6 studies. Final evaluation of the study content showed a result of 5 highly relevant studies (“spot on”) in each of the groups, which suggests a more focused search strategy through the application of AI. The final scores were 49 and 39 points out of 60 for AI groups 1 and 2, respectively, and 46 points for the control group. Data from all relevant studies was extracted, structured, and divided by search teams. All data are presented and summarized in a descriptive manner in Multimedia Appendix 6.

## Discussion

This study was, to our knowledge, the first experiment in the medical field using a science hackathon format and an AI tool to perform a literature search. Both AI groups showed similar quantitative results, but the quality differed between these groups which lead to a difference in their total scores, with a score of 27 points for AI group 1 and a score of 19 points for AI group 2. The control group showed a similar degree of quality as AI group 1 with 25 points awarded, but only 10 of the 46 studies found were categorized as related to the field. AI has been an expanding research area in the last decade, and AI programs have been implemented in various fields, including the medical practice [14]. However, little research has been done on the topic of applying this technology to facilitate literature research. Conventional literature search engines like Google Scholar and PubMed are able to perform key word searches as well as full text searches to some extent, but their ability to recognize similarities is limited to search phrases, available publication titles, or abstract contents [15]. AI, as used by IRIS.AI, has the potential to “understand” the posed research question as well as the screened scientific articles, thus providing the opportunity to filter out the relevant data more effectively. According to the 2015 report [16] of the International Association of Scientific, Technical, and Medical Publishers, approximately 2.5 million scientific papers are published per year, and there are approximately 28,000 active scholarly peer-reviewed research journals. Therefore, a more effective way to perform literature research is highly relevant. In this study, experienced scientific professionals were asked to perform a comprehensive literature review on the question of AR in surgery by applying the artificial search engine of IRIS.AI. When evaluating search results, we focused on the quantity of the relevant articles as well the quality of the results, following the recommendations found in existing literature for the comparison of literature search engines [15,17,18]. Although all participating teams were able to retrieve relevant data on the topic, the results indicate that the application of AI has the potential to focus a scientific search more directly on relevant data and decrease the time necessary to screen the retrieved articles for the actual information needed. Both AI groups showed a significant difference in quality of the detected results, but the AI technology did not provide an increase in search quality. Our study, however, does have some limitations to be considered. First, although all teams were comprised of experts with similar backgrounds, individual differences in know-how and skill can never be entirely excluded, and might pose a bias since only a small sample size of three teams was provided. Second, the qualitative evaluation of search results and the categorization of “relevant” results performed by three judges of high expertise in the relevant fields has the potential to be subjectively biased, and individual preference of the judges in the evaluation can never be entirely excluded. However, to our knowledge, no completely unbiased tool is available. Third, all participants had no previous contact with the IRIS.AI search engine, whereas extensive experience in literature review was present in all participants. Therefore, pre-existing individual preference for certain search engines and a high degree of familiarity in their use were potential biases. Last, although it provides access to most major research databases, the full text access to some databases is not available for the IRIS.AI tool, which poses a potential barrier for the retrieval of relevant information.

This study found a lot of relevant research data on the proposed topic of the development of an adaptive AR system for surgeons to teach surgical residents how to perform a surgical procedure, which makes the format of a science hackathon a useful research tool to gain a fast and effective overview of the literature available for a certain topic.

AI technology is a promising approach to facilitate literature research and comparable to current conventional search engines. In this study, the application of AI technology lead to a more focused literature search without, however, a significant improvement of search results. The format of a scientific hackathon is an efficient tool for literature research, and provides scientists a faster method to gather relevant literature compared to conventional review methods.

## Appendix

Multimedia Appendix 1 Event invitation used in the recruitment process.

Multimedia Appendix 2 Team composition and evaluation of literature search results of all teams.

Multimedia Appendix 3 Report form for literature search results.

Multimedia Appendix 4 Problem statement and definition of research task issued to all participants.

Multimedia Appendix 5 Scoring sheet used in the evaluation of team results.

Multimedia Appendix 6 Summary of search results divided by search teams.

Multimedia Appendix 7 CONSORT-eHEALTH checklist (v 1.6.1).

## Abbreviations

AI: artificial intelligence
AR: augmented reality
IT: information technology
PRISMA: Preferred Reporting Items for Systematic Reviews and Meta-Analysis
VR: virtual reality
