# Supplementary material for: Use of Artificial Intelligence for Medical Literature Search: Randomized Controlled Trial Using the Hackathon Format
Source: Interact J Med Res. 2020 Mar 30;9(1):e16606. doi: 10.2196/16606 (PMC7154940; doi:10.2196/16606)
Supplement: Multimedia Appendix 1 [file ijmr_v9i1e16606_app1.pdf]

Dear Ladies and Gentleman,

We are very happy to welcome you to the **first IRIS.AI Scithon™Event of the medical field**, investigating the topic of virtual and augmented reality in surgery with the help of the IRIS.AI science assistant!

Virtual and Augmented Reality are new cutting edge technologies, with a high potential for application in the medical field. Besides usage for diagnostic imaging applications and the facilitation of minimally invasive procedures, **the implementation of these technologies could substantially improve medical education**, particularly when it comes to teaching practical tasks. In spite of all the technological advances in the medical field, the **nature of surgical training has only marginally changed in the last decades.** Although a variety of dummies and practice models are available, most training is done directly on the patient. While a number of studies and projects have been performed on this topic, more research and development work is necessary to routinely implement those technologies into medical education. To investigate the most promising approach and the questions, that still have to be answered in order to further the implementation of Virtual and Augmented Reality, intensive literature research is necessary. Mapping out the research landscape around a project however, is very time consuming and can often be frustrating as **the sheer size of published data is often nearly impossible to read and understand** much less to be put in context. IRIS.AI created an artificial intelligence technology to help researchers overcome this problem. **The IRIS.AI science assistant is an artificial intelligence technology, which can read scientific papers and to some extent understand their content.** Using this technology allows researchers to overcome the limitations of a human brain when it comes to screening vast amounts of data and find scientific data faster and more efficiently. The Division of Urotechnology, University Medical Center Freiburg, in cooperation with **the Stryker Leibinger GmbH & Co. KG**, **intends with your help to evaluate the IRIS.AI technology for the usage in medical research as well as to map out the research landscape regarding virtual and augmented reality in surgery**.

We are looking forward to a very productive event!

Arkadiusz Miernik

Priv.-Doz. Dr. Dr. med. univ. Arkadiusz Miernik, FEBU
