# Supplementary material for: Use of Artificial Intelligence for Medical Literature Search: Randomized Controlled Trial Using the Hackathon Format
Source: Interact J Med Res. 2020 Mar 30;9(1):e16606. doi: 10.2196/16606 (PMC7154940; doi:10.2196/16606)
Supplement: Multimedia Appendix 2 [file ijmr_v9i1e16606_app2.docx]

## Multimedia Appendix 2: Team composition and evaluation of literature search results of all teams.

| **TABLE 1**: | | | |
| --- | --- | --- | --- |
|  | **AI Team 1** | **AI Team 2** | **Control** |
| **Team composition** | | | |
| Number of  Team Members | 5 | 6 | 6 |
| Team Composition | 1 general surgeon,  1 urologist,  1 software engineer,  1 engineer for robotics,  1 cardiologist | 2 biomedical engineers,  2 urologists,  1ENT,  1 neurosurgeon | 2 biomedical engineers,  1 orthopedic surgeon,  1 general surgeon,  1 neurosurgeon,  1 software engineer |
| **Qualitative result^1^** | | | |
| Quality of the  overview given through the  found data | 10 | 5 | 5 |
| Overall scientific quality of found studies | 8 | 7 | 10 |
| How “up to date” is  the found research | 9 | 7 | 10 |
| **Quantitative result^2^** | | | |
| Found scientific studies as listed by the team | 13 | 15 | 46 |
| Number of papers related to the field  (related) | 10 | 8 | 10 |
| Number of papers containing a relevant approach to the posed question  (relevant) | 7 | 7 | 6 |
| Number of “spot on” papers related to the topic  (highest relevance) | 5 | 5 | 5 |
| **Score in final evaluation (max. Score 60)** | 49 | 39 | 46 |
| *^1^ Quality of search results was assessed and graded: Score ranging from 1 (lowest) to 10 (highest);*  *^2^ One point was awarded for every found study, a maximum of 10 articles was considered for every category* | | | |
