# Supplementary material for: Use of Artificial Intelligence for Medical Literature Search: Randomized Controlled Trial Using the Hackathon Format
Source: Interact J Med Res. 2020 Mar 30;9(1):e16606. doi: 10.2196/16606 (PMC7154940; doi:10.2196/16606)
Supplement: Multimedia Appendix 3 [file ijmr_v9i1e16606_app3.docx]

| Research type facet | Contribution type facet | Paper title | Paper conclusions | Paper ID / URL |
| --- | --- | --- | --- | --- |
| ***What aspect of research are papers about?*** | ***What specific element do papers relate to?*** | ***Please provide the full paper title*** | ***Please provide the paper’s key conclusion*** | ***Please provide a unique paper ID or URL*** |
| Select below one of: validation research, evaluation research, solution proposal, philosophical paper, opinion paper, experience paper, other. | Select below one of: related to a methodology, related to a metric, related to a prototype, related to a process/model, related to a theory, related to an intervention, other. | Insert full title below. | Insert your take on the paper’s key take-aways below. | Insert unique ID below. |
|  |  |  |  |  |
|  |  |  |  |  |
|  |  |  |  |  |
|  |  |  |  |  |
|  |  |  |  |  |
|  |  |  |  |  |
|  |  |  |  |  |

**Multimedia Appendix 3:** Report form for literature search results.
