# Supplementary material for: Use of Artificial Intelligence for Medical Literature Search: Randomized Controlled Trial Using the Hackathon Format
Source: Interact J Med Res. 2020 Mar 30;9(1):e16606. doi: 10.2196/16606 (PMC7154940; doi:10.2196/16606)
Supplement: Multimedia Appendix 4 [file ijmr_v9i1e16606_app4.docx]

**Multimedia Appendix 4:** Problem statement and definition of research task issued to all participants.

In spite of all the technological advances in the medical field, the nature of surgical training has only marginally changed in the last decades. While a variety of dummies and training models are available, currently, most surgical training takes place while performing an intended real life procedure. In order to change this, an augmented reality system, that guides the operating surgeon step by step through a surgical procedure by recognizing the progress of the procedure and the deformation of anatomy is potential beneficial for surgeon and patient alike. There are some important question and technical issues that have to be solved in order to develop a “ready to use” system for the everyday utilization in the operation theatre.

**(i)**The recognition “reality”:

The system needs to be able to recognize deformable human anatomy. Without the application of any artificial markers this is challenging and requires a high amount of computing power. In addition, tracking of surgical instruments and hands of assistant and surgeon is crucial as well. Both of these sensory inputs have to be compiled by the system in order to detect the current state of the operation. This way it can give feedback to the surgeon about the anatomical structures in his field of vision and the next steps that have to be performed.

**(ii)**Augmented reality hardware needs to be light and OR capable, providing a non-distracting heads on display. The information has to be displayed in a way that does not alter the reception of reality; otherwise this could cause a significant risk to the patient. In addition, the system needs to be voice or gesture guided, since the surgeon needs to have both hands free for the surgical procedure. Voice guidance is a particularly difficult task in a noise environment such as the operation theatre.

**(iii)**Pedagogical concept for teaching a complicated surgical task through an AR environment needs to be as efficient as a human teacher. A surgical procedure depicts a complicated task comprised of many different steps that have to be conducted in a certain order to obtain the expected result. While the main steps of any surgical procedure are well defined, descriptions or pedagogical concepts in a sufficiently detailed form are necessary in order to be converted in an AR “step-by-step” instruction.

How can the technical limitations be addressed and how much technical development is needed to overcome these limitations?

What are the technological capabilities of current systems?

Which surgical AR applications have been tested in research and which are commercially available?

Can you come up with a combination of currently available technical solutions to develop a function demonstrator for the above mentioned AR system?
