# Supplementary material for: Use of Artificial Intelligence for Medical Literature Search: Randomized Controlled Trial Using the Hackathon Format
Source: Interact J Med Res. 2020 Mar 30;9(1):e16606. doi: 10.2196/16606 (PMC7154940; doi:10.2196/16606)
Supplement: Multimedia Appendix 5 [file ijmr_v9i1e16606_app5.pdf]

Multimedia Appendix 5: Scoring sheet used in the evaluation of team results.

Qualitative			
Overview - How well did the team manage to explore the overview of the problem? Are there essential parts that are missing? Are there essentials parts that they got wrong? Do they cover all main approaches? Do they address all main challenges? (Max Score 10)			
Findings - Are there interesting findings in the results? If yes what is the quality of the findings - are they well described, are they well supported by research, etc. (Max Score 10)			
Conclusion - Is the conclusion following latest trends in research? Is it well supported by research? Does suggest future research activities? To what extend the proposed future activities follow the trends and are well-motivated? (Max Score 10)			
Quantitative			
Number of related papers to the field (10 are required) (Max Score 10)			
Number of identified relevant approaches (Max Score 10)			
Number of “spot on” papers (surprisingly interesting) (Max Score 10)			
**Total score out of 60:**			
