# Supplementary material for: Use of Artificial Intelligence for Medical Literature Search: Randomized Controlled Trial Using the Hackathon Format
Source: Interact J Med Res. 2020 Mar 30;9(1):e16606. doi: 10.2196/16606 (PMC7154940; doi:10.2196/16606)
Supplement: Multimedia Appendix 6 [file ijmr_v9i1e16606_app6.docx]

**Multimedia Appendix 6:** Summary of search results divided by search teams.

# Team 1

The first team, using the IRIS.AI search tool, listed 13 relevant papers on their score sheet, which were all judged as related to the proposed topic. The quality of the found studies was ranked very high, with a total score of 27. Multiple studies applying AR in surgical fields were detected, showing that AR can provide an accurate visual representation of anatomical structures and is a helpful tool during surgical procedures [1-6]. Specifically, AR was described in the literature as a possibility to offer a highly realistic surgical training experience [7]. Current capabilities of various AR systems go as far as providing real-time augmentation of moving anatomical structures as well as heterogeneous tissues[8, 9], which has been investigated in various fields including ophthalmological, vascular and laparoscopic surgery[1, 5, 10, 11]. Using a combination of surgical navigation platforms, intraoperative imaging and 3D visualization, current technologies can provide the necessary capabilities to construct the proposed adaptive AR system. However, multiple limitations of the currently available technologies exist and were identified in the literature review, defining the potential areas of research necessary to make an AR system ready for clinical use. The main challenges included the available display devices[6], which are still heavy and not suitable for long-term use, the tracking of deformable objects[9], which requires high computational power and is often error-prone and of course the automatic recognition of the surgical workflow[2]. Further challenges are the management of occlusions in object tracking as well as the tracking of instruments in general[2].

## Team 2

The second team supported by artificial intelligence listed 15 relevant papers of which only 8 were found as related to the field by the judges. Quality of the ranked articles was awarded with 19 points. The literature review performed by this team, followed a problem oriented strategy, focusing directly on researching the challenges proposed by the scientific question and on potential solutions available in the literature. Regarding perception of reality and tracking of deformable objects, available technology was detected, that technically addresses the challenges of an adaptive AR system for intraoperative guidance, but the necessity of high computational power and the limited reliability are still challenges that need to be addressed before a clinical use is possible [12-14]. However, instrument tracking technology is already advanced and can potentially be applied in its current state[15] and the technology of processing preoperative imaging for the construction of 3D patient models for intra-operative display also exists in an applicable way[14, 16]. Addressing the difficulty of automatically analyzing and modelling a surgical workflow, different approaches have been proposed, which are able to provide a satisfactory reliability[17-19] and can pose as a base for further developmental work. Multiple studies have addressed the application of AR interfaces for communicating procedural tasks and proven more effective than previous concepts using conventional media[19, 20], but specific research on the concept of instructing surgical procedures through an AR application was not found. A combination of technologies that provide the features necessary to build a “ready to use” adaptive AR system for intraoperative application was not found in the literature [21], but in conclusion research data on all key aspects is available, that can pose as a foundation for further developmental work.

## Control group

The control group listed 46 identified scientific studies the highest number of results, but only 10 were found to be related to the topic. Quality of the studies was graded with a total of 25 points. This group provided a very broad selection of literature, providing an overview of current capabilities of existing technologies for intraoperative support of the operating surgeon as well as limitations and problems, necessary to be addressed when implementing the proposed system into clinical practice. A clear focus on addressing the proposed questions was not visible, which lead to only 10 articles being selected as relevant. Multiple scientific articles about currently available AR systems for intraoperative support were found, covering intraoperative AR navigation [22], AR enhanced robotic surgery [23-28], support systems for intraoperative malignancy detection[23] and surgical skill evaluation [29]. Also, AR technology for surgical skill evaluation and training has already been tested and applied[30-33]. Furthermore, research data on existing pedagogical concepts for simulation medical procedures was found [31, 34], providing a base for conceptual development of an approach for the proposed AR system. Commercially available display systems, which have already been applied to medical applications include Project Tango, Microsoft Holo Lens, Google Glass, Oculus Rift and SimSurgery[35], however all systems are still limited in their applicability to an everyday clinical use[36]. Context aware anatomical detection and tracking of surgical instruments as well as hand tracking and gesture recognition[28, 37-43] are technologies, that are currently under an ongoing development, but need further refinement and improvement to be applicable for the intended purpose of implementation in an adaptive AR support system for intraoperative surgical guidance. Especially the computing power for automated data annotation between the real time high-resolution imaging and the intraoperative navigation to bridge the gap to preoperative diagnostic data is currently not possible to be provided in an everyday operation room setup. A combination of preoperative mapping and segmentation of existing imaging data, combined with intraoperative AR presentation of this data in combination with a state pf the art surgical navigation system can potentially form a base for the development of the proposed system using existing technologies. Real time imaging data can be integrated in the AR system and enriched with the annotated imaging data as well as the information from the surgical navigation system, however very high computational power will be needed with still limited reliability of the resulting AR guidance system.

**References**

1. Davis L, Hamza-Lup FG, Daly J, Ha Y, Frolich S, Meyer C, et al., editors. Application of augmented reality to visualizing anatomical airways. Helmet-and Head-Mounted Displays VII; 2002: International Society for Optics and Photonics.

2. Khor WS, Baker B, Amin K, Chan A, Patel K, Wong J. Augmented and virtual reality in surgery-the digital surgical environment: applications, limitations and legal pitfalls. Ann Transl Med. 2016 Dec;4(23):454. PMID: 28090510. doi: 10.21037/atm.2016.12.23.

3. Mekni M, Lemieux A. Augmented reality: Applications, challenges and future trends. Applied Computational Science. 2014:205-14.

4. Ota D, Loftin B, Saito T, Lea R, Keller J. Virtual reality in surgical education. Comput Biol Med. 1995 Mar;25(2):127-37. PMID: 7554831.

5. Quellec G, Charriere K, Lamard M, Droueche Z, Roux C, Cochener B, et al. Real-time recognition of surgical tasks in eye surgery videos. Medical image analysis. 2014;18(3):579-90.

6. Sielhorst T, Feuerstein M, Navab N. Advanced medical displays: A literature review of augmented reality. Journal of Display Technology. 2008;4(4):451-67.

7. Kamphuis C, Barsom E, Schijven M, Christoph N. Augmented reality in medical education? Perspect Med Educ. 2014 Sep;3(4):300-11. PMID: 24464832. doi: 10.1007/s40037-013-0107-7.

8. Collins T, Bartoli A, Bourdel N, Canis M, editors. Robust, real-time, dense and deformable 3D organ tracking in laparoscopic videos. International Conference on Medical Image Computing and Computer-Assisted Intervention; 2016: Springer.

9. Argotti Y, Davis L, Outters V, Rolland JP. Dynamic superimposition of synthetic objects on rigid and simple-deformable real objects. Computers & Graphics. 2002;26(6):919-30.

10. Cano AM, Gayá F, Lamata P, Sánchez-González P, Gómez EJ, editors. Laparoscopic tool tracking method for augmented reality surgical applications. International Symposium on Biomedical Simulation; 2008: Springer.

11. Haouchine N, Dequidt J, Peterlik I, Kerrien E, Berger M-O, Cotin S, editors. Image-guided simulation of heterogeneous tissue deformation for augmented reality during hepatic surgery. Mixed and Augmented Reality (ISMAR), 2013 IEEE International Symposium on; 2013: IEEE.

12. Maier-Hein L, Mountney P, Bartoli A, Elhawary H, Elson D, Groch A, et al. Optical techniques for 3D surface reconstruction in computer-assisted laparoscopic surgery. Medical image analysis. 2013;17(8):974-96.

13. Van Krevelen D, Poelman R. A survey of augmented reality technologies, applications and limitations. International journal of virtual reality. 2010;9(2):1.

14. Zhou SK. Medical image recognition, segmentation and parsing: machine learning and multiple object approaches: Academic Press; 2015. ISBN: 0128026766.

15. Sahu M, Moerman D, Mewes P, Mountney P, Rose G. Instrument state recognition and tracking for effective control of robotized laparoscopic systems. International Journal of Mechanical Engineering and Robotics Research. 2016;5(1):33.

16. Heimann T, Meinzer H-P. Statistical shape models for 3D medical image segmentation: a review. Medical image analysis. 2009;13(4):543-63.

17. Lalys F, Jannin P. Surgical process modelling: a review. International journal of computer assisted radiology and surgery. 2014;9(3):495-511.

18. Lalys F, Riffaud L, Bouget D, Jannin P, editors. An application-dependent framework for the recognition of high-level surgical tasks in the OR. International Conference on Medical Image Computing and Computer-Assisted Intervention; 2011: Springer.

19. Neumuth T, Jannin P, Strauss G, Meixensberger J, Burgert O. Validation of knowledge acquisition for surgical process models. Journal of the American Medical Informatics Association. 2009;16(1):72-80.

20. Henderson SJ. Augmented reality interfaces for procedural tasks: Columbia University; 2011. ISBN: 1124611959.

21. Bernhardt S, Nicolau SA, Soler L, Doignon C. The status of augmented reality in laparoscopic surgery as of 2016. Medical image analysis. 2017;37:66-90.

22. Watanabe E, Satoh M, Konno T, Hirai M, Yamaguchi T. The trans-visible navigator: a see-through neuronavigation system using augmented reality. World neurosurgery. 2016;87:399-405.

23. Lamata P, Ali W, Cano A, Cornella J, Declerck J, Elle OJ, et al. Augmented reality for minimally invasive surgery: overview and some recent advances. Augmented Reality: InTech; 2010.

24. Hongo K, Kobayashi S, Kakizawa Y, Koyama J-i, Goto T, Okudera H, et al. NeuRobot: telecontrolled micromanipulator system for minimally invasive microneurosurgery—preliminary results. Neurosurgery. 2002;51(4):985-8.

25. Pessaux P, Diana M, Soler L, Piardi T, Mutter D, Marescaux J. Towards cybernetic surgery: robotic and augmented reality-assisted liver segmentectomy. Langenbeck's archives of surgery. 2015;400(3):381-5.

26. Whittaker G, Aydin A, Raison N, Kum F, Challacombe B, Khan MS, et al. Validation of the RobotiX mentor robotic surgery simulator. Journal of endourology. 2016;30(3):338-46.

27. Molliqaj G, Schatlo B, Alaid A, Solomiichuk V, Rohde V, Schaller K, et al. Accuracy of robot-guided versus freehand fluoroscopy-assisted pedicle screw insertion in thoracolumbar spinal surgery. Neurosurgical focus. 2017;42(5):E14.

28. Rieke N, Tan DJ, Tombari F, Vizcaíno JP, di San Filippo CA, Eslami A, et al., editors. Real-time online adaption for robust instrument tracking and pose estimation. International Conference on Medical Image Computing and Computer-Assisted Intervention; 2016: Springer.

29. Reiley CE, Lin HC, Yuh DD, Hager GD. Review of methods for objective surgical skill evaluation. Surgical endoscopy. 2011;25(2):356-66.

30. Cope AC, Bezemer J, Kneebone R, Lingard L. ‘You see?’Teaching and learning how to interpret visual cues during surgery. Medical education. 2015;49(11):1103-16.

31. Christlein V, Ghesu FC, Würfl T, Maier A, Isensee F, Neher P, et al., editors. Tutorial: Deep Learning. Bildverarbeitung für die Medizin 2017: Algorithmen-Systeme-Anwendungen Proceedings des Workshops vom 12 bis 14 März 2017 in Heidelberg; 2017: Springer-Verlag.

32. Yuen SC-Y, Yaoyuneyong G, Johnson E. Augmented reality: An overview and five directions for AR in education. Journal of Educational Technology Development and Exchange (JETDE). 2011;4(1):11.

33. Berry M, Hellström M, Göthlin J, Reznick R, Lönn L. Endovascular training with animals versus virtual reality systems: an economic analysis. Journal of Vascular and Interventional Radiology. 2008;19(2):233-8.

34. Bréaud J, Chevallier D, Benizri E, Fournier J-P, Carles M, Delotte J, et al. The place of simulation in the surgical resident curriculum. The pedagogic program of the Nice Medical School simulation center. Journal of visceral surgery. 2012;149(1):e52-e60.

35. Guha D, Alotaibi NM, Nguyen N, Gupta S, McFaul C, Yang VX. Augmented reality in neurosurgery: a review of current concepts and emerging applications. Canadian Journal of Neurological Sciences. 2017;44(3):235-45.

36. Madhavan K, Kolcun JPG, Chieng LO, Wang MY. Augmented-reality integrated robotics in neurosurgery: are we there yet? Neurosurgical focus. 2017;42(5):E3.

37. Gao Y, Shen D, editors. Context-aware anatomical landmark detection: application to deformable model initialization in prostate CT images. International Workshop on Machine Learning in Medical Imaging; 2014: Springer.

38. Laina I, Rieke N, Rupprecht C, Vizcaíno JP, Eslami A, Tombari F, et al., editors. Concurrent segmentation and localization for tracking of surgical instruments. International Conference on Medical Image Computing and Computer-Assisted Intervention; 2017: Springer.

39. Oikonomidis I, Kyriazis N, Argyros AA, editors. Efficient model-based 3D tracking of hand articulations using Kinect. BmVC; 2011.

40. Keskin C, Kıraç F, Kara YE, Akarun L. Real time hand pose estimation using depth sensors. Consumer depth cameras for computer vision: Springer; 2013. p. 119-37.

41. Sridhar S, Rhodin H, Seidel H-P, Oulasvirta A, Theobalt C, editors. Real-time hand tracking using a sum of anisotropic gaussians model. 3D Vision (3DV), 2014 2nd International Conference on; 2014: IEEE.

42. Sridhar S, Mueller F, Zollhöfer M, Casas D, Oulasvirta A, Theobalt C, editors. Real-time joint tracking of a hand manipulating an object from rgb-d input. European Conference on Computer Vision; 2016: Springer.

43. Sun X, Byrns S, Cheng I, Zheng B, Basu A. Smart sensor-based motion detection system for hand movement training in open surgery. Journal of medical systems. 2017;41(2):24.
